# Reconstruction of Water-Filled Pipe Ultrasonic Guided Wave Signals in the Distance Domain by Orthogonal Matching Pursuit Based on Dispersion and Multi-Mode

**DOI:** 10.3390/s23218683

**Published:** 2023-10-24

**Authors:** Yuemin Wang, Binghui Tang, Ruqing Gong, Fan Zhou, Ang Chen

**Affiliations:** College of Power Engineering, Naval University of Engineering, Wuhan 430033, China; ym99wang@163.com (Y.W.); aoi97s@163.com (R.G.); fan113zhou@163.com (F.Z.); chen8ang1@126.com (A.C.)

**Keywords:** ultrasonic guided wave, orthogonal matching pursuit, dispersion compensation, time-distance mapping

## Abstract

Ultrasonic guided waves (UGWs) in water-filled pipes are subject to more severe dispersion and attenuation than vacant pipes, posing significant challenges for defect identification and localization. To this end, a novel sparse signal decomposition method called orthogonal matching pursuit based on dispersion and multi-mode (DMOMP) was proposed, which utilizes the second-order asymptotic solution of dispersion curves and the conversion characteristics of asymmetric UGWs in the defect contact stage to reconstruct the dispersive signals and converts the time-domain dispersive signals to distance-domain non-dispersive signals by dispersion compensated time-distance mapping. The synthesized simulation results indicate that DMOMP not only exhibits higher reconstruction accuracy compared to OMP, but also reveals more accurate and stable mode recognition and localization compared to DOMP, which only considers the dispersion under perturbation and noise. In addition, the UGW testing experimental results of water-filled pipes verify the effectiveness of DMOMP, the localization accuracies of three feature signals (defct 1, defct 2 and end echo) with DMOMP are 99.10%, 98.72% and 98.36%, respectively, and the average localization accuracy of DMOMP is as high as 98.73%.

## 1. Introduction

Ultrasonic guided waves (UGWs) have gained substantial attention in nondestructive testing (NDT) [1,2] and structural health monitoring (SHM) [3,4] for the low attenuation and high propagation velocity, which are widely adopted in the detection of pipes [5], strands [6], laminates [7], rails [8], bones [9], etc. Notably, considering the ability of UGW testing to characterize the pipe defects that may affect current and future performance, it has also been applied to fluid-filled pipes [10], underground pipes [11], underwater pipes [12], coated pipes [13] and other pipeline systems that take into account the operating situation.

In terms of circumferential energy distribution, axial UGWs can be divided into axisymmetric and non-axisymmetric modes, the former including longitudinal L(0,*m*) and torsional T(0,*m*), and the latter being flexural F(*n*,*m*), where *n* and *m* denote circumferential order and mode [14]. Nevertheless, with the inherent characteristics of UGWs, such as dispersion and multi-mode, which are manifested in that the UGW velocity varies with the frequency and multiple modes of UGWs that exist at a given frequency [15,16], non-target UGWs (non-axisymmetric modes) other than the target UGW (axisymmetric mode) will emerge, which seriously interfere with the determination of the waveguide health status in the form of coherent noise [17].

With the aim of extracting meaningful feature signals from the contaminated UGW signals, the sparse signal decomposition methods, whereby a signal is decomposed into a linear combination of functions that are selected from an over-complete redundant dictionary, have gradually attracted great interest in UGW testing [18,19]. Wang et al. [20] proposed an improved matching pursuit (MP)-based temperature and load compensation method for UGW signals, which has verified excellent effects under the influence of temperature and load variations. Zheng et al. [21] introduced orthogonal matching pursuit (OMP) into the dispersive radon transform and achieved the extraction and separation of UGW modes. Xu et al. [22] detected the weld defect from strong background noises by introducing the split-augmented Lagrangian shrinkage algorithm to basis pursuit (BP). Zhang et al. [23] employed the multiple sparse Bayesian learning (M-SBL) strategy for the damage imaging of composite laminates.

A well-constructed dictionary comprising atoms is the significant factor for determining the convergence speed and accuracy of the sparse signal decomposition method. Numerous atoms in different analytic forms have been chosen to construct dictionaries, such as gabor [24], chirplet [25] and Hanning window-modulated sinusoidal signals [26]. However, these dictionaries do not work well in representing the dispersive UGW signals, which are excited by the sinusoidal signal modulated by the Hanning window, and for this reason, other dictionaries have been proposed. Cai et al. [27] proposed linearly dispersive signal construction (LDSC) for the dispersion compensation of Lamb waves, considering the complexity of absolute wavenumber determination. Lian et al. [28] constructed a dictionary composed of atoms with truncated Nakagami functions to approximate the overlapping UGW echoes. Chen et al. [29] studied the nonlinear Hanning-windowed Chirplet (NHWC) model to eliminate the inconsistency between the known Chirplet model and practical situations. However, the original purpose of the above dictionary was only to maximize the reconstruction accuracy of the sparse signal decomposition, and it could not directly reveal the corresponding modes and propagation distances of the dispersive signals, which are quite often the most concerning contents of the UGW testing.

When localization analysis of the defect signal is required, the dispersion characteristics remain to be employed to determine the corresponding modes and propagation distance. Xu et al. [30] built a dispersive dictionary utilizing the dispersion curves of UGWs to decompose the dispersive UGW signal and the non-dispersive dictionary for the dispersion compensation of the reconstructed signal. Kim et al. [31] constructed a dictionary based on an asymptotic solution of the dispersion relation for Lamb waves in tone burst wave packets, which included parametric information, such as the propagation time delay, dispersion extent and phase. Rostami et al. [32] used the finite element method to predict the wave packets propagating along the waveguide, which have a maximum resemblance with real UGW signals. Nevertheless, the waveguides targeted in the above studies were plate-like structures and vacant pipes, for which their effectiveness in dealing with water-filled pipes with more serious dispersion is unknown, and considering only the dispersion of the target mode UGW may be insufficient for the sparse reconstruction of the UGW signals and localization of defects.

In order to balance the reconstruction and localization accuracy as much as possible, an orthogonal matching pursuit based on dispersion and multi-mode (DMOMP) is proposed, which utilizes the second-order asymptotic solution of dispersion curves and the conversion characteristics of asymmetric UGWs in the defect contact stage, which was not considered in previous studies, to reconstruct the dispersive signals and convert the time-domain dispersive signals to distance-domain non-dispersive signals by dispersion compensated time-distance mapping to visualize the position of defects. In addition, considering the effects of water on the pipe UGW, such as energy attenuation and mode coupling, there has been no sparse signal decomposition study on water-filled pipes to the best of the authors’ knowledge.

The structure of this paper is as follows: the UGW characteristics, including water-filled pipe, are analyzed in Section 2; the dispersion compensated time-distance mapping is introduced in Section 3; the signal sparse decomposition with the proposed method is progressively studied through dispersive and non-dispersive atoms (Section 4), DOMP (Section 5) and DMOMP (Section 6); Section 7 evaluates the processing effects of DMOMP on experimental UGW signals and Section 8 concludes the paper.

## 2. UGW Characteristics of Water-Filled Pipe

**Figure 1:**
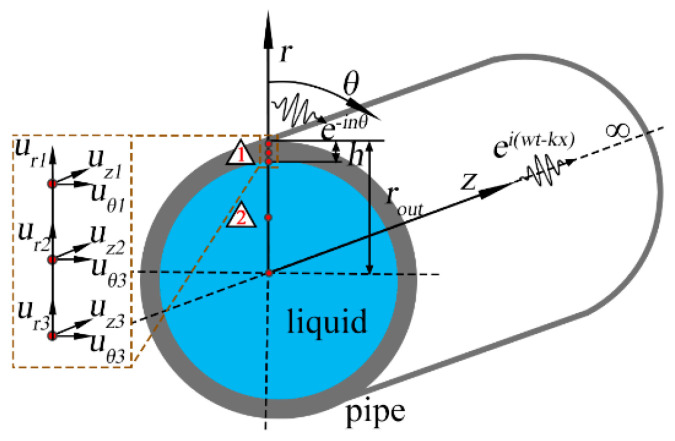
Schematic diagram of liquid-filled pipe in the column coordinate system.

Figure 1 displays the schematic diagram of liquid-filled pipe in the cylindrical coordinate system, and the space-time harmonic function *e^i^*^(*ωt−kx−nθ*)^ is assumed to represent the propagation of UGW, where *i* is the imaginary unit, *ω* is the angular frequency, *t* is the time, *k* is the wavenumber, *n* is the circumferential order, *r*, *θ* and *z* denote the radial, circumferential and axial [33]. In the semi-analytical finite element (SAFE) method, the finite element can only be performed in the *r* direction and divided into two elements according to the material, and the displacement *u* of each node is divided into *u_r_*, *u_θ_* and *u_z_*.

Considering the coupling phenomenon between the solid and liquid, the dispersion characteristics of liquid-filled pipelines can be solved by SAFE [34,35], which were obtained by the AXISAFE open-source program proposed by Kalkowski in this paper [36]. Given *n* and *ω*, the eigenvalue problem of SAFE can be solved, of which the eigenvalue and eigenvector correspond to the wavenumber *k* and the wave structure. Take the real part of the eigenvalue *k_Re_*, and the dispersion curves of phase velocity *cp* = *ω*/*k_Re_* and group velocity *cg* = ∂*ω*/∂*k_Re_* can be calculated [36,37].

A carbon steel pipe filled with water was analyzed using AXISAFE, and the model parameters were defined as follows. (1) Material properties: the pipe and water properties are given in Table 1, where *ρ* is the density, *E* is the Young’s modulus, *μ* is the Poisson’s ratio, *K* is the bulk modulus and the symbol “-” indicates no value; (2) Cross-section dimensions: both the pipe and water need to define the internal radius *r_in_* and wall thickness *h* of the cross-section, which are also shown in Table 1; (3) Frequency vector: the frequency vector is the linear spacing vector from 1 kHz to 200 kHz (5000 points); (4) Element sets: the radial coordinates are [*r_in_*,*r_in_* + *h*], and the element types of the pipe and the water are SLAX6 and ALAX6, respectively. (5) Circumferential order: the order of the axisymmetric UGW is 0, and the order of the non-axisymmetric UGW is an integer greater than 0.

### 2.1. Dispersion

**Figure 2:**
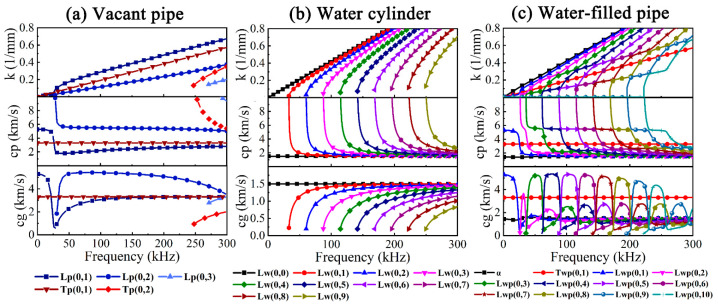
Dispersion curves of axisymmetric UGWs: (**a**) vacant pipe, (**b**) water cylinder and (**c**) water-filled pipe.

The axisymmetric UGWs dispersion curves of the vacant pipe, water cylinder and water-filled pipe are depicted in Figure 2. There are three L modes and two T modes in the vacant pipe, which exhibit different dispersion characteristics as the frequency varies from 0 kHz to 300 kHz. Non-dispersion is exhibited in Tp(0,1) showing constant velocity, yet Lp(0,2) shows non-dispersion between 50 and 150 kHz and severe dispersion between 28 and 50 kHz.

Since water cannot withstand shear, the UGWs in water cylinders have no torsional modes, and only 10 longitudinal modes Lw(0,0–9) exist, as illustrated in Figure 2b. In spite of Lwp(0,*m*) and Twp(0,*m*) in Figure 2c, the *α*-mode can also be observed, which is dominated by radial displacement with dispersion in the low-frequency band and is similar to the Scholte wave with almost no dispersion in the high-frequency band [38,39]. Furthermore, there seems to be no influence of water on Tp(0,1) and Lp(0,1), whereas Lp(0,2) is split into multiple modes Lwp(0,2–10) by Lw(0,1–9), which means that Lwp(0,2–10) can be considered to be the combination of two water cylinder modes and a vacant pipe mode for the mode coupling of UGWs in different media [40].

### 2.2. Multi-Mode

**Figure 3:**
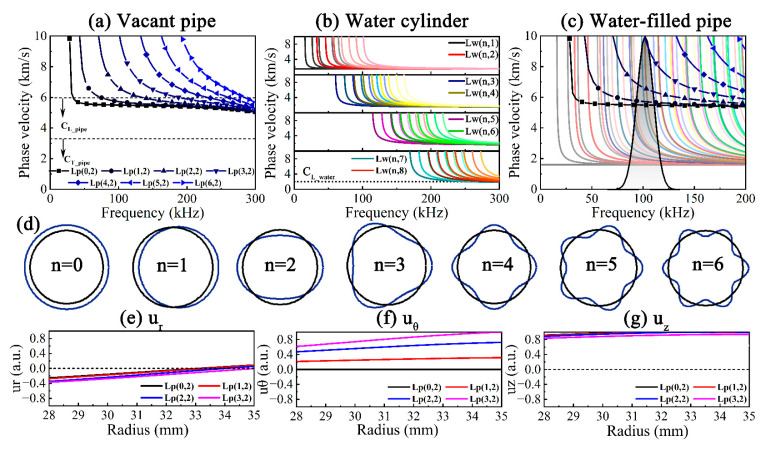
Dispersion curves of non-axisymmetric UGWs: (**a**) vacant pipe, (**b**) water cylinder and (**c**) water-filled pipe, (**d**) mode shapes, (**e**) radial, (**f**) circumferential and (**g**) axial displacement distributions of Lp(*n*,2) at 105 kHz.

For ease of distinction, F(*n*,*m*) can be written in the form of L(*n*,*m*), and the phase velocity dispersion curves of Lp(*n*,2) and Lw(*n*,*m*) are illustrated in Figure 3a,b and the dotted lines show the longitudinal *c_L_* and transverse *c_T_* wave velocities, of which water has no *c_T_* due to its inability to withstand shear. It can be concluded that the cut-off frequencies gradually increase with the increase in *n*, and the dispersion becomes stronger in the meantime. The mode shapes of UGWs are displayed in Figure 3d; the periodic distributions of circumferential displacements related to *n* are shown, embodying more complex scenarios. The dispersion curves of Lp(*n*,2) and Lw(*n*,*m*) can be put together for comparison, as shown in Figure 3c. Lp(0,2) corresponding to Lwp(0,5) at 105 kHz in the non-dispersive band was chosen as the target UGW, using a five-period Hanning window-modulated sinusoidal signal as the excitation, and its spectrum is represented by a black curve with gray gradient filling, as also seen in Figure 3c.

Lp(0,2) is most likely to be converted to Lp(*n*,2) (*n* = 1, 2, 3) because its cut-off frequencies are below the upper bound of the excitation frequency band. It can also be concluded from Figure 3e–g that Lp(*n*,2) at 105 kHz is dominated by axial displacement, and the circumferential displacement becomes significantly larger as *n* increases, while the radial and axial displacements slightly change. In summary, the asymmetric UGWs greatly differ from symmetric UGW in terms of mode shape, displacement distribution, velocity, attenuation and dispersion. In general, UGWs in water-filled pipes exhibit stronger dispersion than in vacant pipes, with shorter non-dispersive frequency bands and more modes existing at the same frequency.

## 3. Dispersion-Compensated Time-Distance Mapping

As the name implies, dispersion-compensated time-distance mapping is the method used to transform a dispersion-compensated UGW signal from the time domain to the distance domain. The dispersion compensation and time-distance mapping are briefly reviewed in this section, and the effects of the dispersion-compensated time-distance mapping are revealed by the synthesized UGW signals.

### 3.1. Dispersion Compensation

Consider a Hanning window modulated sinusoidal signal as the excitation, which is defined by

(1) fet=ht−ht−2πNωc1−cosωctNeiωct

where *h*(*t*) is the Heaviside function, *N* is the number of periods, *ω_c_* is the central angular frequency and the Fourier form of *f_e_*(*t*) is noted as *F_e_*(*ω*). The dispersive UGW signal containing M different orders and modes can be expressed as [33]

(2) $$f_{d}^{}\left(t\right)=\frac{1}{2\pi }\sum_{i=1}^{M}\int_{-\infty }^{\infty }F_{e}\left(\omega \right)e^{i\omega t}e^{-ik_{d}^{i}x_{0}}d\omega$$

where the subscript *d* stands for dispersion. *k_d_* is usually a frequency-dependent curve, and the straight line with a fixed slope is manifested in the non-dispersive *k*. In this way, the non-dispersive *k* can be written as *k_nd_* = *k*_0_ + *k*_1_ (*ω* − *ω_c_*), where *k*_0_ denotes *k_d_* corresponding to *ω_c_* and *k*_1_ denotes the reciprocal of the *cg* corresponding to *ω_c_*.

The dispersion compensation can be applied to *f_d_*(*t*) with the priori knowledge of dispersion, which is expressed as

(3) $$f_{com}^{}\left(t\right)=\frac{1}{2\pi }\sum_{i=1}^{M}\int_{-\infty }^{\infty }F_{e}\left(\omega \right)e^{i\omega t}e^{-ik_{nd}^{i}x_{0}}d\omega$$

### 3.2. Time-Distance Mapping

Provided that the propagation distance *x* is considered as the variable and the time *t* is considered as 0 (the excitation moment), then the time-domain signal can be expressed in the distance domain as [31]

(4) $$f_{com}^{}\left(x\right)=\frac{1}{2\pi }\sum_{i=1}^{M}\int_{-\infty }^{\infty }F_{\mathrm{nd}}\left(\omega \right)e^{ik_{nd}^{i}x}d\omega$$

Considering that *k* and *x* are similar to the Fourier transform pair of *ω* and *t*, *dω* can be expressed in the form of *cg*(*ω*)*dk_d_* by the definition, which is substituted into Equation (5), and utilizing the relationship between *ω* and *k_d_* in the dispersion curve, we obtain

(5) $$f_{com}^{}\left(x\right)=\frac{1}{2\pi }\sum_{i=1}^{M}\int_{-\infty }^{\infty }F_{\mathrm{nd}}\left(\omega \right)cg\left(\omega \right)e^{ik_{nd}^{i}x}dk_{nd}^{i}$$

Let *G*(*k_d_*) equal *F_d_*(*ω*)*cg*(*ω*), Equation (5) can be directly inverse Fourier transformed to obtain the dispersion compensation signal in the distance domain.

### 3.3. Analysis of Synthesized UGW Signals

**Figure 4:**
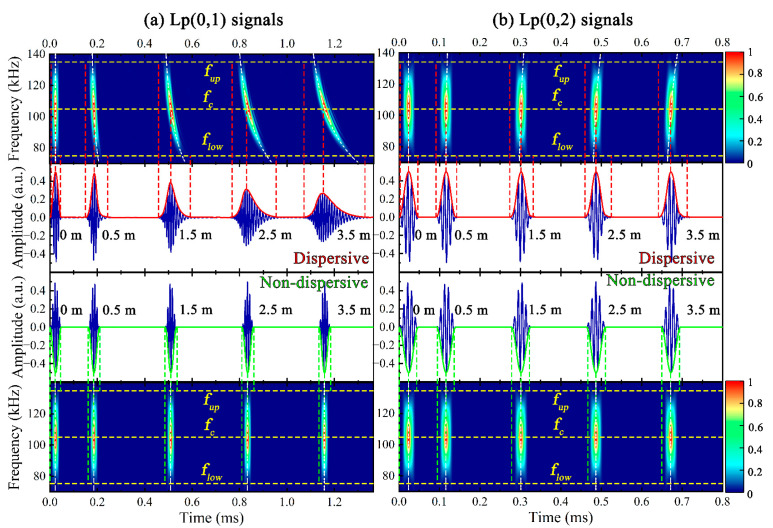
(**a**) Lp(0,1) and (**b**) Lp(0,2) signals at 105 kHz before and after applying dispersion compensation.

The time domain signals of Lp(0,1) and Lp(0,2) at different *x* (0.5 m, 1.5 m, 2.5 m, 3.5 m) are plotted in Figure 4. For ease of observation, the center frequency *f_c_*, the lower *f_low_* and upper *f_up_* frequency boundary of the excitation band are marked with yellow dashed lines in the time-frequency diagram, and the white dashed lines represent the group dispersion curves at the given *x*, which is calculated by *x*/*cg*(*ω*) + 0.5*T*, where *T* is the duration of the excitation signal.

**Figure 5:**
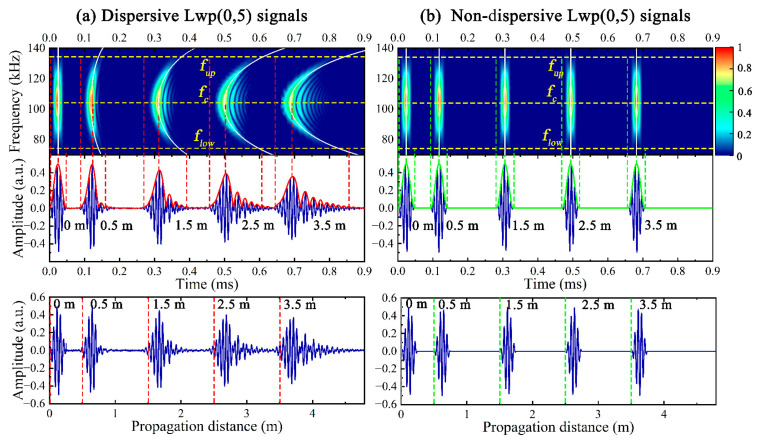
Time-distance mapping results of (**a**) dispersive and (**b**) non-dispersive Lwp(0,5) signals at 105 kHz.

It can be observed that the dispersive Lp(0,1) exhibits significant attenuation and dispersion as the wave packet gradually becomes wider and the amplitude gradually becomes smaller as *x* increases, while the waveform and amplitude of the dispersive Lp(0,2) signal do not noticeably change with *x*. The dispersion-compensated time-distance mapping was performed on the above dispersive signals, the attenuation and waveform deformation could not be observed in the non-dispersive signals, which were consistent with the excitation signal except for the phase and time, and the group velocities of the non-dispersive signals could be considered as the fixed values for the time-frequency wave packets parallel to the frequency axis. Compared with the dispersive Lwp(0,5) signals in Figure 5, the non-dispersive Lwp(0,5) exhibited a better match with the actual *x*, which is favorable to guide the actual UGW testing.

## 4. Dispersive and Non-Dispersive Atoms

Given that *k* dictates the dispersion characteristics, it is an efficient way to expand *k*(*ω*) at *ω_c_* in the Taylor series, which can be called the asymptotic solution. Ignore the high-order terms and take the second-order asymptotic solution, for example [41]

(6) $$k\left(\omega \right)=k_{0}+k_{1}\left(\omega -\omega_{c}\right)+k_{2}{\left(\omega -\omega_{c}\right)}^{2}+o{\left(\omega -\omega_{c}\right)}^{2}$$

where *k*_0_ = *k*(*ω_c_*), *k*_1_ = (d*k*/d*ω*)|*_ωc_* = 1/*cg_c_*, *k*_2_ = (d^2^*k*/d*ω*^2^)|_ωc_/2. The UGW signal propagating to the given distance can be rewritten as

(7) $$f_{d}^{}\left(x,t\right)=\frac{1}{2\pi }\int_{-\infty }^{\infty }F_{e}\left(\omega \right)e^{i\omega t}e^{-i\left[k_{0}+k_{1}\left(\omega -\omega_{c}\right)+k_{2}{\left(\omega -\omega_{c}\right)}^{2}\right]x}d\omega$$

The complete expressions of the dispersive and non-dispersive atoms are written as

(8) $$a_{\Theta =[\omega_{c},N,k_{0},k_{1},k_{2},x,\theta ]}^{d}\left(t\right)=\frac{\xi \left(\Theta \right)}{2\pi }e^{i\left(\omega_{c}t-k_{0}x+\theta \right)}\times \int_{-\infty }^{\infty }\frac{i\omega_{c}^{2}}{\omega \left(\omega_{c}^{2}-N^{2}\omega^{2}\right)}\left(e^{-i2\pi \omega N/\omega_{c}}-1\right)e^{i\left[\left(t-k_{1}x\right)\omega -k_{2}x\omega^{2}\right]}d\omega$$

(9) $$a_{\Theta =[\omega_{c},N,k_{0},k_{1},x]}^{nd}\left(t\right)=\frac{\xi \left(\Theta \right)}{2\pi }e^{i\left(\omega_{c}t-k_{0}x\right)}\times \int_{-\infty }^{\infty }\frac{i\omega_{c}^{2}}{\omega \left(\omega_{c}^{2}-N^{2}\omega^{2}\right)}\left(e^{-i2\pi \omega N/\omega_{c}}-1\right)e^{i\left(t-k_{1}x\right)\omega }d\omega$$

where Θ denotes the atom parameters, $\xi \left(\Theta \right)$ is the coefficient that normalizes the atom *a* to unit 2-norm, that is, ||*a*|| = 1, and *θ* is the additional phase term that takes into account the possible phase changes in the actual UGW testing.

### 4.1. Analysis of UGW Dispersive and Non-Dispersive Atoms

**Figure 6:**
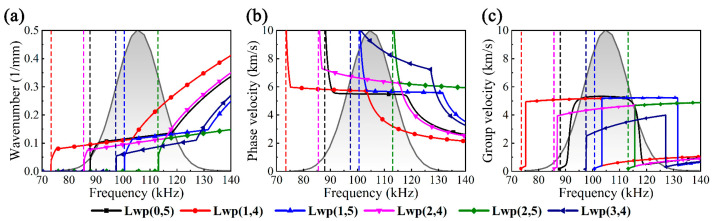
Dispersion curves of water-filled pipe in the excitation band: (**a**) wavenumber, (**b**) phase velocity and (**c**) group velocity.

The multi-mode UGWs consisting of the target and its flexural UGWs were studied to set the stage for the following. Figure 6 depicts the dispersion curves in the excitation band, where the black and white gradient-filled portion of the solid black envelope represents *F_e_*(*ω*) and the vertical dashed lines represent the cut-off frequencies. The most likely flexural UGWs converted from Lwp(0,5) within the excitation band include Lwp(1,4), Lwp(1,5), Lwp(2,4), Lwp(2,5) and Lwp(3,4).

**Figure 7:**
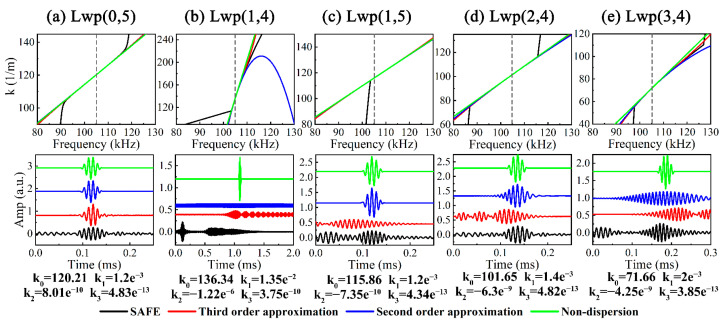
(**a**) Lwp(0,5), (**b**) Lwp(1,4), (**c**) Lwp(1,5), (**d**) Lwp(2,4) and (**e**) Lwp(3,4) signals obtained from SAFE and asymptotic solution at 0.5 m.

The asymptotic solutions of *k* from first to third order were studied in order to represent the above UGWs, where *k*_3_ = (d^3^*k*/d*ω*^3^)|_ωc_/6; the results are indicated in Figure 7. However, the asymptotic solution does not truly reflect the variation of *k* over a wider frequency band and the steep change of *k* near *ω_c_*; i.e., a significant gap between the asymptotic and SAFE solution can be observed in Lwp(1,4), Lwp(1,5) and Lwp(3,4) near *ω_c_*. In addition, it can be observed that third-order asymptotic solution signals exist in the form of multi-wave packets.

In the actual UGW testing of the water-filled pipe, the UGW signals generated by the water cylinder may not have affected the vacant pipe UGW signals as seriously as the theoretical SAFE signal for the attenuation and environmental interference, and it is reasonable to adopt the first- and second-order asymptotic solution signals of a single wave packet as non-dispersive and dispersive atomic representations of the UGW signals.

### 4.2. Effects of Atom Parameters

**Figure 8:**
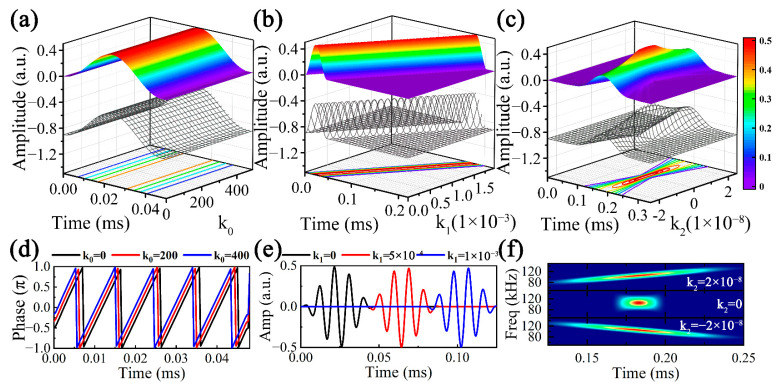
Effects of atom parameters on the time-domain signal envelopes: (**a**) *k*_0_, (**b**) *k*_1_ and (**c**) *k*_2_, (**d**) instantaneous phase variation of different *k*_0_, (**e**) time-domain variation of different *k*_1_, (**f**) time-frequency domain variation of different *k*_2_.

The parameters determining the atoms include *ω_c_*, *N*, *k*_0_, *k*_1_, *k*_2_ and *x*, where *ω_c_* and *N* control the excitation signal, thus the input of *k*_0_, *k*_1_, *k*_2_ and *x* outputs the corresponding atom. As the detailed analysis of the UGW signals at different *x* was included in Section 3.3, the focus of this section is studying the effects of *k*_0_, *k*_1_ and *k*_2_ on the atoms, setting *ω_c_* = 105 kHz, *N* = 5 and *x* = 0.5 m, and the results are illustrated in Figure 8.

Assuming both *k*_1_ and *k*_2_ are zero, the effects of *k*_0_ on the atoms alone are displayed in Figure 8a. It is clear that *k*_0_ does not affect the envelope of the time-domain atoms. However, as can be observed by the instantaneous phases corresponding to different *k*_0_ (0, 200, 400) in Figure 8d, the instantaneous phases of the atoms all periodically and linearly change, and the change of *k*_0_ leads to a time shift in the instantaneous phase.

It can be observed from Figure 8b that the effects of *k*_1_ ∈ [0~2 × 10^−3^] on the envelope of the time-domain atoms, assuming that both *k*_0_ and *k*_2_ are zero, are time-shifted, which is also reflected in the time-domain atom waveforms plotted in Figure 8e. It is found that *k*_1_ can be considered as a time-shifted term. Suppose *k*_0_ = 0 and *k*_1_ = 2 × 10^−3^, *k*_2_ ∈ [−2 × 10^−8^~2 × 10^−8^] has a nonlinear effect on the time-domain atoms. The larger the absolute value of *k*_2_, the wider the wave packet, while the peak moments of the time-domain atoms are constant. Moreover, according to the time-frequency analysis of the atoms corresponding to different *k*_2_ in Figure 8f, it can be concluded that a negative value of *k*_2_ leads to a gradual decrease in the instantaneous frequency with time, while a positive *k*_2_ does the opposite, and the above trends become more pronounced as the absolute value of *k*_2_ increases. Obviously, *k*_2_ is the main reason for the dispersion, and even a very small order of magnitude of *k*_2_ (e.g., 10^−8^) cannot be ignored for the significant effect on the atoms in the time and time-frequency domains.

## 5. Orthogonal Matching Pursuit Based on Dispersion (DOMP)

**Figure 9:**
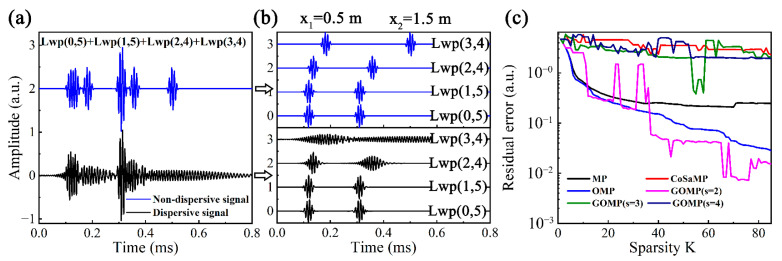
(**a**) Synthesized multi-mode UGW signal and (**b**) its components before and after applying dispersion compensation, (**c**) RESs of OMP and other sparse signal decomposition methods.

It is essential to study the processing effects of OMP before the description of DOMP. Only Lwp(0,5) and its flexural modes are considered, due to the low propagation speed of Lwp(1,4), which would be captured at the lagged moment, so four modes of Lwp(0,5), Lwp(1,5), Lwp(2,4) and Lwp(3,4) are considered to be superimposed. Suppose the above UGWs have two sets of wave packets at *x*_1_ = 0.5 m and *x*_2_ = 1.5 m, respectively, the synthesized signal is shown in Figure 9a, and the components are displayed in Figure 9b. The synthesized signal is decomposed by OMP and other methods, varying the sparsity *K* (1–90) and calculating the residual errors (RESs), and the results are shown in Figure 9c.

The RESs of the above methods show a tendency to decrease with increasing *K*. MP, OMP and GOMP (*s* = 2) converge faster, and their RESs are as low as 2.46 × 10^−1^, 1.99 × 10^−2^ and 8.91 × 10^−3^ when *K* = 90. It can be predicted that when *K* is large enough, the reconstructed signal can be considered to be completely restored to the synthesized signal. Considering the convergence speed and residual stability, OMP is chosen as the sparse signal decomposition method.

### 5.1. Methodology

As the name suggests, DOMP is the OMP based on the dispersion characteristic of UGWs. The inputs of DOMP are the *N_r_* × *N_c_* dispersive and non-dispersive dictionary matrices **D^d^** and **D^nd^**, where $N_{c}\gg N_{r}$, the *N_r_* × 1 received signal **f_r_** and the sparsity *K*. The outputs include the *K* × 1 sparse coefficients ${\hat{A}}_{K}$, the *N_r_* × *K* dispersive and non-dispersive atomic sets $D_{K}^{d}$ and $D_{K}^{nd}$, the reconstructed *N_r_* × 1 dispersive and non-dispersive signals ${\hat{f}}_{r}^{d}$ and ${\hat{f}}_{r}^{nd}$ and the *N_r_* × 1 residual error $r_{K}$.The process of DOMP is represented in pseudocode, as shown in Algorithm 1.
**Algorithm 1:** DOMP.$\mathrm{Initialize}\;r_{0}\leftarrow f_{r}$$,\;\Lambda_{0}\leftarrow \emptyset$$,\;D_{0}^{d}\leftarrow \emptyset$$,\;D_{0}^{nd}\leftarrow \emptyset$ **for** *k* = 1:*K*
**do** $I_{k}\leftarrow \arg\underset{j=1,2,\ldots ,N_{c}}{\max}\left|\langle r_{k-1},a_{j}^{d}\rangle \right|$$,\;\Lambda_{k}\leftarrow \Lambda_{k-1}\cup \left\{I_{k}\right\}$$,\;D_{k}^{d}\leftarrow D_{k-1}^{d}\cup a_{I_{k}}^{d}$$,\;D_{k}^{nd}\leftarrow D_{k-1}^{nd}\cup a_{I_{k}}^{nd}$ ${\hat{A}}_{k}\leftarrow \arg\underset{{\hat{A}}_{k}}{\min}\left\|f_{r}-D_{k}^{d}A_{k}\right\|\leftarrow {\left(D_{k}^{dT}D_{k}^{d}\right)}^{-1}D_{k}^{dT}f_{r}$$,\;r_{k}\leftarrow f_{r}-D_{k}^{d}{\hat{A}}_{k}$ **end for** ${\hat{f}}_{r}^{d}\leftarrow D_{K}^{d}{\hat{A}}_{K}$$,\;{\hat{f}}_{r}^{nd}\leftarrow D_{K}^{nd}{\hat{A}}_{K}$where *k* is the iteration number, **r***_k_* is the residual of the *k*-th iteration, $\Lambda_{k}$ is the index set (column number of the dictionary matrix) of the *k*th iteration, *I_k_* is the index found in the *k*th iteration, $a_{j}^{d}$ is the *j*th column of **D^d^**, *N_r_* × *k* $D_{k}^{d}$ is the set of columns selected from **D^d^** according to $\Lambda_{k}$ and **A***_k_* is the *k* × 1 sparsity factor.

### 5.2. Simulation Results

**Figure 10:**
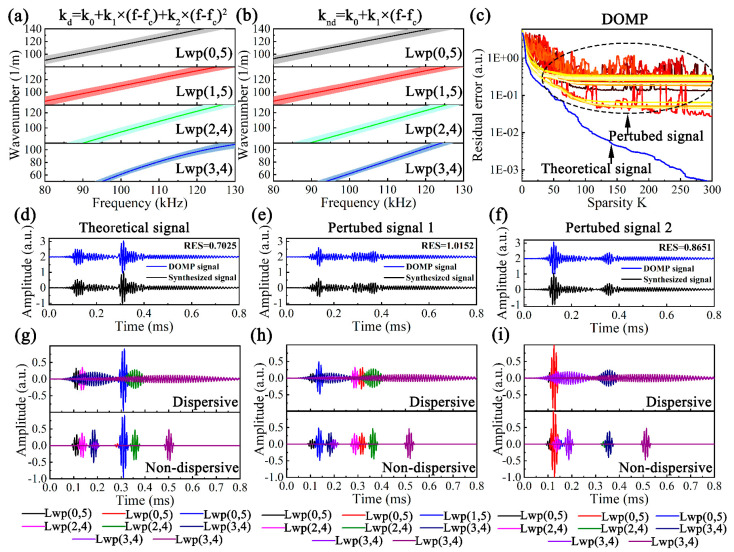
(**a**) Dispersive and (**b**) non-dispersive perturbed *k* of Lwp(0,5), Lwp(1,5), Lwp(2,4) and Lwp(3,4), (**c**) RESs of perturbed signals after applying DOMP, (**d**) theoretical signal, (**e**) perturbed signal 1 and (**f**) perturbed signal 2 before and after applying DOMP and decomposed atoms: (**g**) theoretical signal, (**h**) perturbed signal 1 and (**i**) perturbed signal 2.

The actual physical properties of water-filled pipes inevitably deviate from the theoretical values, resulting in the accurate dispersion curves being difficult to obtain, which means that the DOMP based on inaccurate dispersion characteristics may suffer from error. To this end, the dispersion perturbation analysis of DOMP is crucial. Considering that the asymptotic solution of *k* can be determined by the parameter *k_j_* (*j* = 0, 1, 2), the problem is turned into the perturbation of *k_j_*. Figure 10a,b illustrates the theoretical dispersive and non-dispersive *k* for different modes represented by asymptotic solutions. The dark solid lines represent the theoretical *k* and the light-colored areas represent the variation range of the perturbed *k*. The perturbed parameter ${\hat{k}}_{j}\in \left[\left(1-\delta \right)k_{j}\sim \left(1+\delta \right)k_{j}\right]$, where $j\in \left[0,1,2\right]$ and *δ* are set to 0.05 to make the parameters vary between ±5%.

**D^d^** and **D^nd^** include the dictionaries of all modes, namely **D** = [**D**1, **D**2, **D**3, **D**4]. The parameters of **D***i* can be expressed as $\Theta_{i}=[f_{c},N,{\hat{k}}_{\left(i,0\right)},{\hat{k}}_{\left(i,1\right)},{\hat{k}}_{\left(i,2\right)},x,\theta ]$, where *f_c_* and *N* are 105 kHz and 5, *i* denotes the *i*-th mode, $x\in \left[0_{s}\sim cg\left(f_{c}\right)\left(N_{r}-1\right)/f_{s}\right]$ and $\theta \in \left[0\sim \pi \right]$. Apply DOMP to the theoretical and perturbed signals, the RESs are shown in Figure 10c and the magnitude order of RES of the perturbed signal is basically stable around 10^−1^ after 300 iterations, while that of the theoretical signal is not gradually stable, but continues to decrease to 10^−3^ with iterations.

Given that a large *K* can certainly improve the reconstruction accuracy, it will inevitably bring about over-decomposition. Considering the synthesized signal in Figure 9a includes eight parts, it is reasonable to set *K* to 8. Two perturbed signals from Figure 10c were randomly selected and noted as perturbed signals 1 and 2, respectively. The reconstructed signals after applying DOMP are shown in Figure 10d–f. The DOMP with the theoretical dispersion has the best effect on the theoretical signal (TS) with an RES of 0.7025, while the perturbed signals 1 and 2 (PS1 and PS2) have a slightly worse effect, with RESs of 1.0152 and 0.8651, respectively.

The decomposed dispersive and non-dispersive atoms of DOMP are illustrated in Figure 10g–i, and the modes and *x* of each atom are extracted, which are shown in Table 2. The decomposed atoms are not necessarily the actual components of the synthesized signal. In the case of TS, its corresponding DOMP decomposed results do not match the actual situation, where 3 Lwp(0,5), 2 Lwp(2,4) and 3 Lwp(3,4) signals and the Lwp(1,5) signals are missing, which can be attributed to the serious overlap in the time domain for the similar group velocities of Lwp(0,5) and Lwp(1,5) at 105 kHz. For the perturbed signals, it is also impossible to achieve a one-to-one correspondence between the decomposed atoms and the synthesized signal components. In the case of Lwp(0,5), the *x* errors between PS1 [0.4381 m, 1.5301 m], PS2 [0.4575 m, 0.6509 m] and the actual distances [0.50 m, 1.50 m] are [12.38%, 2.07%] and [8.5%, 56.61%], and such large errors are insufficient to guide the actual UGW defect testing.

## 6. Orthogonal Matching Pursuit Based on Dispersion and Multi-Mode (DMOMP)

It is meaningless to pursue reconstruction accuracy and give up the actual meaning of decomposed atoms. The reconstruction accuracy of DOMP can be improved by increasing the dictionary size and *K*, but at the same time, it may bring the problems of large computational burden and meaningless decomposed atoms, and the correspondence between the decomposed atoms and the actual modes cannot be guaranteed. To this end, DMOMP was proposed, which takes into account the dispersion and mode conversion characteristics of multi-mode UGWs.

### 6.1. Methodology

In the mode conversion period, a part of the target mode UGW may be converted to flexural mode UGWs, which is completed within a very small time scale and it can be understood that their propagation distances are the same. The above properties are equivalent to adding a constraint to the DOMP, which makes the decomposed results more consistent with the actual modes. In addition, it is not necessary to construct a nondispersive dictionary in advance, but to convert the decomposed time-domain dispersive signals to the range-domain non-dispersive signals by using the parameter relationship. The process of DMOMP is represented in pseudocode, as shown in Algorithm 2.
**Algorithm 2:** DMOMP.$\mathrm{Initialize}\;r_{0}\leftarrow f_{r}$$,\;{\hat{f}}_{r}^{d}\leftarrow \emptyset$$,\;{\hat{f}}_{r}^{nd}\leftarrow \emptyset$ $\left[\hat{A}1_{K1},D1_{K1}^{d},\Lambda_{K1}\right]\leftarrow \mathrm{DOMP}\left(D1^{d},f_{r},K1\right)$$,\;\hat{x}1\leftarrow x1\left(\Lambda_{K1}\right)$$,\;lb\leftarrow \left(1-\alpha \right)\hat{x}1$$,\;ub\leftarrow \left(1+\alpha \right)\hat{x}1$ **while** *i* <= *K*1 **do** **for**
*j* = 1:*L* **do** ${\hat{x}}_{j}\leftarrow lb\left(i\right)+\left(j-1\right)/\left(L-1\right)\left[ub\left(i\right)-lb\left(i\right)\right]$$,\;D_{new}^{d}\leftarrow \left[{\hat{D}}_{1}^{d},{\hat{D}}_{2}^{d},\ldots ,{\hat{D}}_{K2}^{d}\right]$ $\left[{\hat{A}}_{j},D_{j}^{d},\Lambda_{j}\right]\leftarrow \mathrm{DOMP}\left(D_{new}^{d},r_{j-1},K2\right)$ **end for** $[RES,k]\leftarrow \min\left\|r\right\|$$,\;{\hat{f}}_{r}^{d}\leftarrow {\hat{f}}_{r}^{d}+D_{k}^{d}{\hat{A}}_{k}$ **end while** $\Theta^{nd}\leftarrow \Theta^{d}$$,\;{\hat{f}}_{r}^{nd}\leftarrow {\hat{f}}_{r}^{d}$$,\;{\hat{f}}_{r}^{nd}(x)\leftarrow {\hat{f}}_{r}^{nd}(t)$

(1) The target mode UGW is dominant in **f_r_**, for which the dictionary **D**1**^d^** and propagation distance set **x**1 can be constructed and $\Lambda_{K1}$ can be obtained by applying DOMP on **f_r_** with the sparsity *K*1. Determine the set $\hat{x}1$ of possible propagation distances for each target mode guide wave based on the coarsely selected $\Lambda_{K1}$, and the range can be expressed by upper **ub** and lower **lb** bounds;
(2) For the *i*-th target mode UGW, traverse ${\hat{x}}_{j}$ according to **lb**(*i*) and **ub**(*i*) (*j* refers to the *j*-th iteration and $j\in \left[1,2,\ldots ,L\right]$), and construct the dictionary $D_{new}^{d}$, which is consistent with the dictionaries of *K*2 mode at the given ${\hat{x}}_{j}$. The residual error r can be obtained by DOMP, taking the index *k* to correspond to the smallest RES ($\left\|r\right\|$), and the decomposed results of *i*-th target mode and its flexural modes are determined, namely ${\hat{A}}_{k}$ and $D_{k}^{d}$;
(3) Repeat step (2) *K*1 times to obtain the reconstructed signals $D_{k}^{d}{\hat{A}}_{k}$ corresponding to *i*-th target mode and its flexural modes, the time-domain reconstructed results ${\hat{f}}_{r}^{d}$ can be obtained by summing. The final distance-domain non-dispersive signal ${\hat{f}}_{r}^{nd}(x)$ can be obtained by the dispersion-compensated time-distance mapping.

### 6.2. Sensitivity of Perturbation

**Figure 11:**
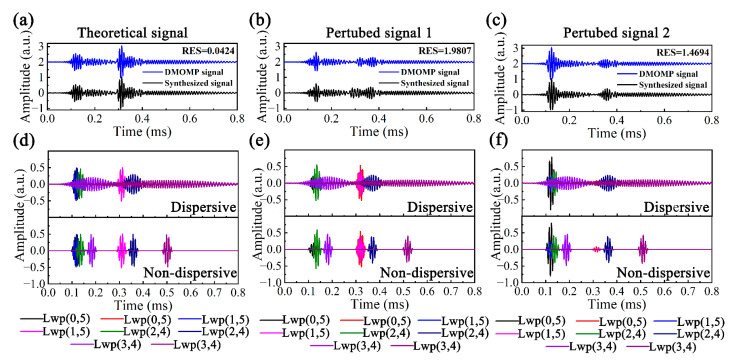
(**a**) Theoretical signal, (**b**) perturbed signal 1 and (**c**) perturbed signal 2 before and after applying DMOMP, and DMOMP atoms: (**d**) theoretical signal, (**e**) perturbed signal 1 and (**f**) perturbed signal 2.

Apply DMOMP to the above signals in Section 5.2, and the reconstruction and decomposed results are shown in Figure 11. Compared with DOMP, the reconstruction accuracy of DMOMP for TS is further improved, the RES is only 0.0424, but the reconstruction accuracy of the PS1 and PS2 becomes lower, at 1.9807 and 1.4694, respectively. Although the DMOMP sacrifices the reconstruction accuracy to some extent, it can be determined that DMOMP signals share the same mode distribution with the unprocessed signals.

**Figure 12:**
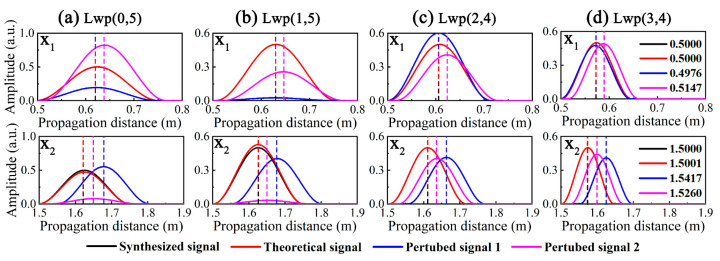
DMOMP decomposed non-dispersive atoms in the distance domain: (**a**) Lwp(0,5), (**b**) Lwp(1,5), (**c**) Lwp(2,4) and (**d**) Lwp(3,4).

The non-dispersive decomposed atoms obtained from DMOMP were transformed in the time-distance domain and the Herbert transform was performed to take the envelopes; the results are shown in Figure 12. From the *cg* dispersion curves of the liquid-filled pipe in Figure. 6, it can be seen that the *cg*s of Lwp(0,5) and Lwp(1,5) are close to each other with the largest values, followed by Lwp(2,4) and Lwp(3,4) with the smallest group velocities, and since *x* is proportional to *cg*, each mode with the same width in the time domain presents a different width in the distance domain.

To clearly illustrate the position of the decomposed atoms of DMOMP in the distance domain, the peak positions of the different signals are indicated by dashed lines. For the wave packets at *x*_1_ = 0.5 m, the propagation distance and amplitude of the theoretical signal before and after processing are the same, which is also reflected by the overlap of the envelope. The propagation distance of the processed PS1 is almost the same as the theoretical value with an error of only 0.48%, and the error of the processed perturbed signal 2 is slightly larger at 2.94%. For the wave packets at *x*_2_ = 1.5 m, the dispersion phenomenon is more serious and the processing effect of DMOMP becomes slightly worse, especially for the perturbed signals, and their errors are 2.78% and 1.73%, but they are still lower than those of DOMP.

### 6.3. Sensitivity to Noise

**Figure 13:**
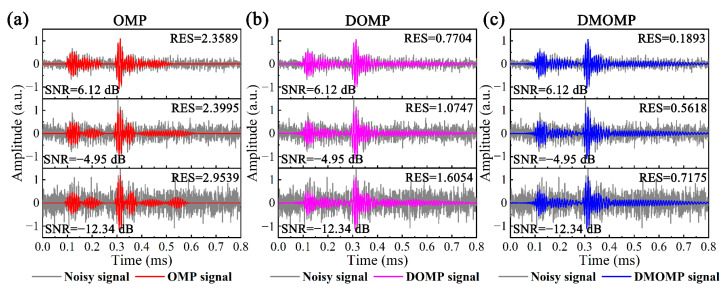
Noisy signals before and after applying (**a**) OMP, (**b**) DOMP and (**c**) DMOMP.

It is essential to analyze the noise sensitivity for the influence of non-coherent noise induced by environmental factors. The noisy signals composed of theoretical signal and noise were obtained, where the noise level is represented by the signal-to-noise ratio (SNR), and the reconstructed signals of OMP, DOMP and DMOMP are displayed in Figure 13. As the noise level increases, the SNR becomes lower, and the RES gradually becomes larger, indicating the certain influence of noise on the reconstruction accuracy. When the noise level is large (SNR = −12.34 dB), the theoretical signal is almost completely drowned out by the noise and it is impossible to discriminate each mode, while the noisy signal components can all be revealed to some extent by sparse signal decomposition. Compared with OMP and DOMP, DMOMP has the highest reconstruction accuracy, with an average reduction of 80.93% and 57.39% in RES, indicating the advantages of DMOMP in noisy signal reconstruction.

Take Lwp(0,5), the dominant part of the noisy signal, as an example, to illustrate *x* and its error with the theoretical value after the decomposition by the above methods, as shown in Table 3, the dictionary of OMP is the Hanning window modulated sinusoidal signals, so its decomposed atoms do not contain mode information, and *x* can only be estimated by *x* = *cg_c_* × *t*. Although the DOMP decomposition atoms contain mode information, the *cg* difference between Lwp(0,5) and Lwp(1,5) is small, and the phenomenon of mistaking Lwp(0,5) for Lwp(1,5) may occur during the decomposition; for example, when SNR = −4.95 dB, the Lwp(0,5) wave packet at *x*_1_ = 0.5 m is not decomposed, but two Lwp(0,5) wave packets at *x*_2_ = 1.5 m appear. DMOMP can reveal both the mode and *x* information, and *x* of Lwp(0,5) has a very small error in terms of the theoretical value. Discarding the outliers, the average error of DMOMP is reduced by 3.35% and 2.94% compared to OMP and DOMP, and the decomposition results of the propagation distance are less affected by noise, even when the SNR is as low as −12.34 dB, the average error only increases by 0.11%.

## 7. Experimental UGW Testing Results

**Figure 14:**
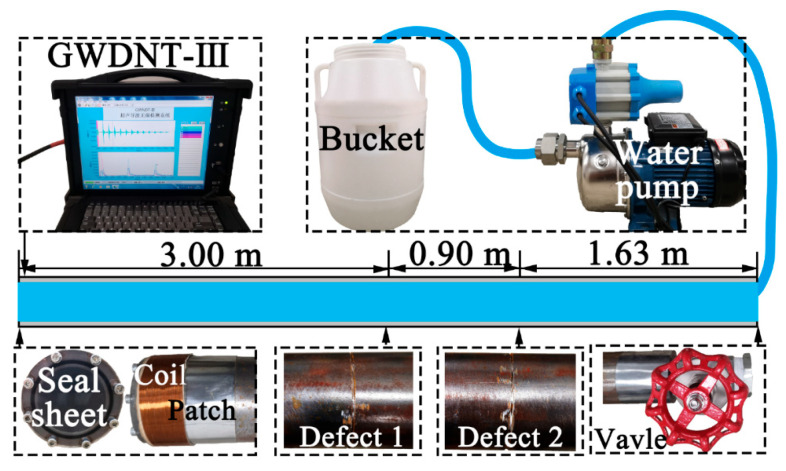
Schematic diagram of the UGW testing platform for water-filled pipe.

The self-made GWNDT-III instrument based on the magnetostrictive principle was used for UGW testing of the above water-filled pipe, which is self-excited and self-accepting with a coil the width of which is controlled by the half-wavelength of Lwp(0,5) at 105 kHz. The excitation signal was the five-period Hanning window modulated sinusoidal signal of 5 V, with 100 excitations, a 200 ms excitation interval, a 1 MHz sampling frequency and a 3 ms sampling time. The UGW testing platform of the water-filled pipe is illustrated in Figure 14, a transparent acrylic plate was installed at the excitation/receiving end for the purpose of sealing and observing the liquid level. A ball valve was installed at the other end and connected to the pump and bucket to ensure the pipe was completely filled with water. The length of the pipe was 5.53 m. The defects were considered to be set at 3.00 m and 3.90 m. The width and depth of the defects were 1 mm and the circumferential length was expressed as the angle (90–360°).

**Figure 15:**
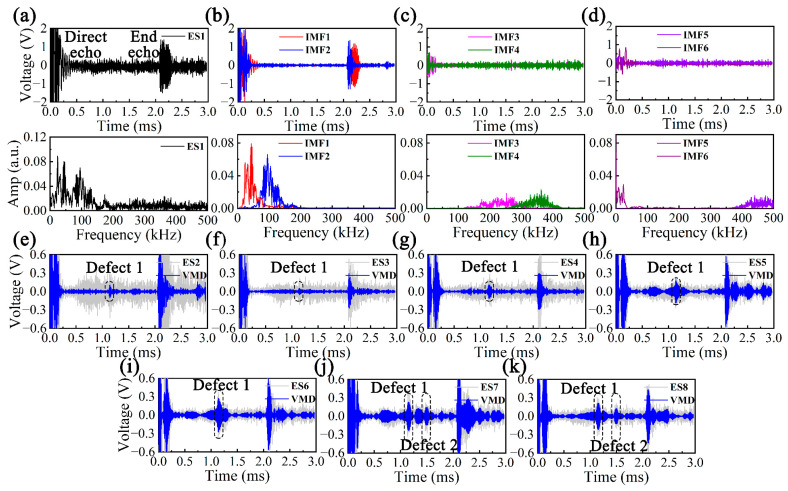
(**a**) ES1 in time and frequency domains, (**b**–**d**) IMFs of ES1 after applying VMD, (**e**) ES2, (**f**) ES3, (**g**) ES4, (**h**) ES5, (**i**) ES6, (**j**) ES7 and (**k**) ES8 before and after applying VMD.

Firstly, the defect-free vacant pipe was tested and ES1 was obtained, as shown in Figure 15a. Within 0–3 ms, two obvious wave packets emerged, corresponding to the direct echo and the pipe end echo, and the noise at a high level could also be observed, resulting in the low SNR. It could be noticed, by observing the spectrum, that ES1 included components in the lower frequency band due to the dispersion and multi-modes, in addition to the components around 105 kHz. In this regard, it was decomposed using variational mode decomposition (VMD) to obtain IMFs, as shown in Figure 15b–d [42]. It was found that the center frequency of IMF2 was around 105 kHz, which best reflected the actual condition of the pipe under test, and has a high SNR, which was more favorable for the subsequent analysis.

Defect 1 (90°) was artificially set for the defect-free vacant pipe and noted as ES2. Defect 1 (90°, 180°, 270° and 360°) was set on the surface of the water-filled pipe and noted as ES3-6. Defect 1 (360°) and defect 2 (180° and 270°) were set and noted as ES7-8. Similarly, ES2-8 was processed by VMD, taking the IMFs with the *f_c_* around 105 kHz, and the results are displayed in Figure 15e–k. It could be concluded that the water resulted in serious attenuation and dispersion, which were reflected in the reduction of the end-echo amplitude and increase in noise level. In addition, as the circumferential length and number of defects increased, the coherent noise level became higher, which seriously interfered with the extraction of useful information in the signal. Therefore, VMD can reflect the state of the pipe to some extent, but it is somewhat unrealistic to achieve the accurate identification and localization of defects.

**Figure 16:**
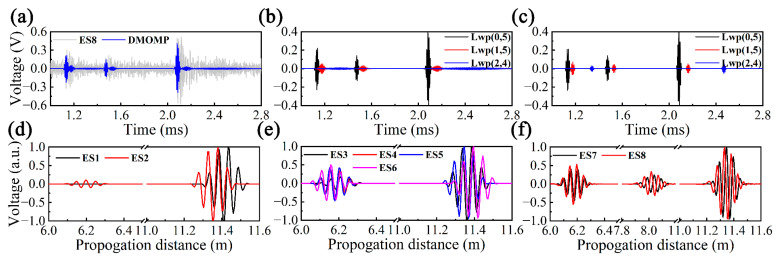
(**a**) ES8 before and after applying DMOMP, (**b**) dispersive and (**c**) non-dispersive atoms of ES8 after applying DMOMP, dispersion compensated time-distance mapping results of (**d**) ES1-2, (**e**) ES3-6 and (**f**) ES7-8.

The effects of DMOMP on the experimental UGW signals are illustrated using ES8 as an example, as illustrated in Figure 16a–c. The reconstruction results consisting of nine atoms intuitively visualize defect 1, defect 2 and end echo and present a high SNR. It can be observed from the decomposition of ES8 that Lwp(1,5) and Lwp(2,4) are most likely to occur due to the mode conversion of Lwp(0,5). The non-dispersive atoms obtained by DMOMP were converted into the distance domain, and the distance domain signals of ES1-8 were finally obtained, as depicted in Figure 16d–f. Obviously, the distance domain signals are able to visualize the propagation distance while preserving the feature signals, such as defects and the pipe end, which is beneficial for defect localization.

The propagation distances of ES1-8 after applying DMOMP were extracted and are listed in Table 4, where *x*_1_, *x*_2_ and *x*_3_ correspond to the locations of defect 1, defect 2 and the pipe end, respectively. Regardless of whether the pipe is water-filled or defective, DMOMP presents excellent localization ability, and the average errors of DMOMP for ES1-8 are only 0.0270 m, 0.0499 m and 0.0905 m compared to the actual positions of *x*_1_, *x*_2_ and *x*_3_ (3.000 m, 3.9000 m and 5.5300 m), with accuracies of 99.10%, 98.72% and 98.36%. Although the localization accuracy of DMOMP slightly decreases as the propagation distance of pipe features becomes larger, DMOMP can accurately identify and locate pipe features.

Although the DMOMP is able to convert time-domain dispersive ultrasonic guided wave signals into distance-domain non-dispersive ultrasonic guided wave signals with high localization accuracy, the running time is long due to the need to construct a dictionary, and the localization accuracy of the DMOMP may be degraded when the actual situation differs from the theory due to the utilization of the theoretical dispersion characteristics. For this reason, future research requires improving the efficiency of the sparse signal decomposition method and considering the actual pipeline dispersion characteristics.

## 8. Conclusions

In order to balance the reconstruction accuracy and practical meaning of reconstructed signals as much as possible and intuitively and accurately realize the defect localization in water-filled pipes, a novel sparse signal decomposition method called orthogonal matching pursuit based on dispersion and multi-mode (DMOMP) is proposed; it can reveal information of modes and propagation distances that OMP cannot directly obtain, and it is also more accurate than DOMP results.

DMOMP utilizes the second-order asymptotic solution of dispersion curves and the conversion characteristics of asymmetric UGWs in the defect contact stage to reconstruct the dispersive signals, and converts the time-domain dispersive signals to the distance-domain non-dispersive signals by dispersion compensated time-distance mapping. The synthesized simulation results indicate that DMOMP achieves a good balance between reconstruction accuracy and actual signal meaning despite perturbation and noise, the average localization error of DMOMP is reduced by 3.35% and 2.94% compared to OMP and DOMP and it is less affected by noise; even when the SNR is as low as −12.34 dB, the average localization error only increases by 0.11%. The experimental results of water-filled pipe UGW testing verify the effectiveness of DMOMP, the localization accuracies of three feature signals (defct 1, defct 2 and the end echo) with DMOMP are 99.10%, 98.72% and 98.36%, respectively, and the average localization accuracy of DMOMP is as high as 98.73%.

## Figures and Tables

**Table 1:** Material properties and cross-section dimensions of the pipe and water [36].

Material	*ρ* (kg/m^3^)	*E* (Pa)	*μ*	*K* (Pa)	*r_in_* (mm)	*h* (mm)
Pipe	7800	2.17 × 10^11^	0.28	-	28	7
Water	1000	-	-	2.25 × 10^9^	0	28

**Table 2:** Modes and propagation distances of DOMP decomposed atoms for theoretical signals, perturbed signals 1 and 2.

	TS			DOMP (TS)			DOMP (PS1)		DOMP (PS2)	
	Mode (Lwp)	*x* (m)	Amp (a.u.)	Mode (Lwp)	*x* (m)	Amp (a.u.)	Mode (Lwp)	*x* (m)	Mode (Lwp)	*x* (m)
1	(0,5)	0.50	0.48	(0,5)	0.4627	0.33	(0,5)	0.4381	(0,5)	0.4575
2	(0,5)	1.50	0.47	(0,5)	1.3808	0.06	(0,5)	1.5301	(0,5)	0.5360
3	(1,5)	0.50	0.49	(0,5)	1.4986	0.91	(1,5)	0.5952	(0,5)	0.6509
4	(1,5)	1.50	0.47	(2,4)	0.5043	0.35	(2,4)	1.3819	(2,4)	0.5061
5	(2,4)	0.50	0.46	(2,4)	1.4998	0.28	(2,4)	1.5238	(2,4)	1.4189
6	(2,4)	1.50	0.30	(3,4)	0.5043	0.21	(3,4)	0.4997	(3,4)	1.4898
7	(3,4)	0.50	0.21	(3,4)	0.5260	0.05	(3,4)	0.5518	(3,4)	0.5119
8	(3,4)	1.50	0.12	(3,4)	1.5015	0.11	(3,4)	1.5467	(3,4)	1.5331

**Table 3:** Propagation distances and errors of Lwp(0,5) in the noisy signals after the decomposition of OMP, DOMP and DMOMP.

		SNR = Inf dB		SNR = 6.12 dB		SNR = −4.95 dB		SNR = −12.34 dB	
		Value (a.u.)	Error (%)	Value (a.u.)	Error (%)	Value (a.u.)	Error (%)	Value (a.u.)	Error (%)
OMP	*x*_1_	0.4834	3.3200	0.4877	2.4661	0.4663	6.7299	0.4577	8.4600
	*x*_2_	1.5092	0.6133	1.51897	1.2653	1.5322	2.1543	1.5430	2.8636
DOMP	*x*_1_	0.4627	7.4600	0.4621	7.5800	1.3755	175.1000	0.4873	2.5400
	*x*_2_	1.4986	0.0933	1.4986	0.0933	1.4986	0.0933	1.4986	0.0933
DMOMP	*x*_1_	0.5000	0.0000	0.5001	0.0200	0.5001	0.0200	0.5011	0.2200
	*x*_2_	1.5001	0.0067	1.5001	0.0067	1.5001	0.0067	1.5001	0.0067

**Table 4:** Propagation distances of ES1-8 after applying DMOMP.

	ES1	ES2	ES3	ES4	ES5	ES6	ES7	ES8	Actual Value	Accuracy
*x*_1_ (m)	-	3.0348	3.0409	3.0146	3.0267	3.0247	3.0247	3.0227	3.0000	99.10%
*x*_2_ (m)	-	-		-	-	-	3.9530	3.9469	3.9000	98.72%
*x*_3_ (m)	5.6436	5.6146	5.6188	5.6354	5.6105	5.6105	5.6105	5.6064	5.5300	98.36%

## Data Availability

The data is privacy to us, and we cannot provide it.
